# Cognitive image, affective image, cultural dimensions, and conative image: A new conceptual framework

**DOI:** 10.3389/fpsyg.2022.935814

**Published:** 2022-08-02

**Authors:** Shaohua Yang, Salmi Mohd Isa, Yiyue Yao, Jinyuan Xia, Danping Liu

**Affiliations:** ^1^School of Business, Anhui University of Technology, Maanshan, China; ^2^Graduate School of Business, Universiti Sains Malaysia, Penang, Malaysia; ^3^Guangxi University Xingjian College of Science and Liberal Arts, Nanning, China; ^4^School of International Education, Anhui University of Technology, Maanshan, China; ^5^School of Management, Xihua University, Chengdu, China; ^6^Research Institute of International Economics and Management, Xihua University, Chengdu, China

**Keywords:** destination image, cross cultural studies, tourist behavior, Hofstede's cultural dimensions, cognitive-affective-conative model, individualism, uncertainty avoidance

## Abstract

Destination image is essential to tourists' loyalty and has been discussed in length among researchers and marketers in the tourism industry for decades. Based on a literature review, the destination image model, including cognitive image, affective image, and conative image, has been firmly established as an acceptable means to gain an understanding of tourists' behavior toward revisiting and recommendations. The understanding of the moderating role of cultural constructs is still unclear, especially in cross-cultural travel behavior. Therefore, this conceptual paper proposes an integrated model of cognitive-affective-conative image that includes the constructs of individualism and uncertainty avoidance. Based on the underpinning theories and empirical studies, this paper proposes affective image potentially mediates the correlation between cognitive image and conative image. This model also incorporated individualism and uncertainty as potential moderating effects between affective image and conative image. By integrating individualism and uncertainty avoidance into the theoretical model from the perspective of tourism, this paper contributes to a more comprehensive understanding of the influence of travel behavior on emerging tourism marketing.

## Introduction

Tourism destination is a highly competitive component of the tourism industry. Due to the advanced and frequent diversification of destination image, there is an increase in overall competitive advantages in the international tourism marketplace (Kester and Croce, 2011). With the accomplishment of garnering tourists' interest to visit a particular destination, the capability of identifying tourists' reasons for their destination choices is becoming even more crucial. Furthermore, destination image has been utilized to capture tourists' perception of a destination (Crompton, 1979), indicating the comprehensive imagery a tourist holds. Moreover, destination image provides those in the industry with an opportunity to appropriately design and deliver effective promotional strategies for a destination product (Um and Crompton, 1990; Hsu et al., 2010). Destination image has also gained increasing attention from scholars in the tourism field as it is essential in tourists Kester decision-making processes (i.e., Beerli and Martín, 2004a; Tseng et al., 2015; Chen et al., 2016; Yang et al., 2021a, 2022). To be specific, destination image has been investigated in several studies as a factor in tourists' behavioral intentions to visit and revisit a destination (Assaker et al., 2011; Cheng and Lu, 2013; Chew and Jahari, 2014; e.g., Alvarez and Campo, 2014; Whang et al., 2016; Stylos and Bellou, 2019; Yang et al., 2021a,c, 2022).

Notably, controversial topics are presented in examining the compositions of the destination image used to predict tourists' behaviors toward a holistic destination image. Accordingly, a coherent body of studies adopted the typology of Garnter's (1993) models of destination images such as cognitive, affective, and conative images. The above-mentioned models were examined primarily in terms of the direct or indirect impacts of destination image compositions on tourists' behaviors (Agapito et al., 2013; Chew and Jahari, 2014; Papadimitriou et al., 2015, 2018; Tosun et al., 2015; Lindblom et al., 2018; Stylidis et al., 2020b). These studies indicate that tourists' intentions to revisit and provide feedback/ recommendations are due to the destination image model. In gaining an improved understanding of this matter, scholars confirmed that the affective component mediates the effect between the cognitive and conative components (Agapito et al., 2013).

Although destination components were examined (Stylos et al., 2017), the affective and cognitive images were the only foci of previous research, without taking the conative image into account (Zhang et al., 2014). Moreover, only recent studies attach great importance to the conative images to delineate tourists' intentions to revisit and recommend a destination (Stylos et al., 2016). However, Bigné et al. (2009) pointed out the research lacuna in the dominant image dimensions that shape tourists' prospective behavior intentions, thus underscoring that the importance of the three components remains unexplored. Therefore, this paper aims to depict the comparative importance of all destination image components in predicting tourists' conative behaviors, specifically their intentions to revisit and recommend a destination. This relative importance was directly and indirectly delineated from the cross-cultural travel perspective. In fulfilling this objective, a cross-cultural approach was implemented, where two cultural constructs were added to a destination image model.

In respect of cross-cultural travel relevance, another two constructs that may be crucial predictors of tourists' behavioral intentions are uncertainty avoidance and individualism. These constructs were conceptualized by Hofstede's theory of cultural dimension (1980). Hofstede's cultural multidimensions theory was incorporated into many studies as a key construct in tourism research due to the increasing internationalization of the tourism market (Crotts, 2004; Litvin et al., 2004; Reisinger and Mavondo, 2005; Matzler et al., 2016; Seo et al., 2018; Yang et al., 2021b). These studies aimed to illustrate the importance of cultural background in tourists' experiences. They also elaborated on the reasons for the cultural background to be a major factor in tourists' behavioral intentions. Specifically, British tourists had a higher level of loyalty in terms of revisiting a destination (Kozak, 2001). Comparatively, Russian tourists possessed low levels of loyalty and high eagerness to visit more parts of the world. This finding could be explained by how the Russian culture, which possesses a low degree of uncertainty avoidance, is predisposed to endure a high level of personal risk (Hofstede, 1980, 2001) and has a high level of individualism (Naumov and Puffer, 2000).

In exploring the cross-cultural approach to tourism, it was agreed that Hofstede's multidimensional framework was the most appropriate predictor of tourists' behavioral intentions (Money and Crotts, 2003). Besides providing an early foundation, Hofstede's cultural multidimensional framework remains most influential and universally applied by scholars studying from cultural perspectives (Soares et al., 2007) and it takes a predominant place in cross-cultural research conducted among tourists (Reisinger and Turner, 2003; Ng et al., 2007; Qian et al., 2018). Although Hofstede's cultural framework was the most widely adopted model in business literature, not all cultural dimensions of this model are suitable to examine tourists' behaviors (Money and Crotts, 2003; i.e., Crotts and Pizam, 2003). Previous literature showed that the inclusion of all dimensions to categorize travellers' behaviors can result in cultural bias and prejudices (Huang and Crotts, 2019). For instance, tourism and hospitality scholars proposed that only uncertainty avoidance and individualism were the most relevant cultural dimensions in the context of cross-cultural travel among international tourists (Money and Crotts, 2003; Crotts, 2004; Litvin and Kar, 2004; Litvin et al., 2004; Meng, 2010; Seo et al., 2018; Yang et al., 2022). The application of these two typologies at the individual level is suitable in this conceptual paper because tourists with different values might be identified based on cultural dimensions. Patterson et al. (2006) put forward the idea that individual cultural traits provide greater explanatory power than nationality.

Since destination image is a crucial factor in the tourism domain (Tse and Tung, 2022), the destination image model (i.e., cognitive, affective, and conative) employed in extant studies have tried to unveil the connections between exogenous and endogenous variables in the tourism context (Agapito et al., 2013; Chew and Jahari, 2014; Fu et al., 2016; Stylos et al., 2017; Woosnam et al., 2020). As the above-mentioned studies in tourism literature always studied these variables in isolation, research integrating these notions with culture-related factors in a unified nomological network is rare. More importantly, none of the existing studies offer insights into the moderating role of individualism and uncertainty avoidance from the perspective of destination image model. Therefore, this article intends to propose the effect of cognitive, affective, and conative images and to explore how individualism and uncertainty avoidance possibly moderate the interactions between the affective and conative elements.

Several contributions are made by this conceptual paper. First, this article provides insights into how crucial the aforementioned components are for tourists to make a decision and for the establishment of a model for cross-cultural travel (i.e., international traveling). Second, it presents an argument regarding the important role played by the combined effects of the image components, individualism, and uncertainty avoidance in the prediction of conative images. Third, apart from new insights that complemented previous findings, this study also elaborates on the moderating roles of individualism and uncertainty in tourists' decision-making processes, specifically in terms of how possibly these image components shape tourists' behavioral intentions to revisit a destination. Last, this article has practical implications such as useful recommendations and destination marketing strategies for tourism stakeholders.

## Literature review

### Theoretical background

In proposing one seminal theory on destination image, Gartner (1993) developed a hierarchical cause and effect model based on three aspects, namely, cognitive, affective, and conative images. This model was also supported by several researchers as it was used to gain an understanding of tourists' behavioral intentions (Pike and Ryan, 2004; Tasci and Gartner, 2007; Tasci et al., 2007). Furthermore, it was in line with the study by Boulding (1956) who elaborated that an image consisted of an individual's knowledge and thoughts about an object (cognitive), their perceptions of it (affective), and their actions toward this information (conative). Unlike image construct and vacation destinations, the cognitive (also known as intellectual/perceptual) component is linked with an individual's conception and acknowledgment of potential features of the destination. Meanwhile, the affective component is linked to the evaluation stage, which primarily focuses on the individuals' feelings related to their destination (Gartner, 1993; Baloglu and Brinberg, 1997; Baloglu and McCleary, 1999; Beerli and Martín, 2004a,b). Meanwhile, the conative image refers to action, such as tourists' actual conduct or intentions to revisit and recommend destinations (Gartner, 1993; Bigné et al., 2001; Pike and Ryan, 2004; Konecnik and Gartner, 2007; Tasci and Gartner, 2007; Tasci et al., 2007). In the context of tourism, the conative image refers to a traveler's actions of sharing positive feedback (Baker and Crompton, 2000), provided they have any intentions of doing so.

The three components of destination image contribute to an understanding of the construction of a global image, which is assumed to be more significant than some of its components. This construction is applied by consumers to make more easier decisions (Echtner and Ritchie, 1993; Stern and Krakover, 1993; Baloglu and McCleary, 1999; Beerli and Martín, 2004a,b). These components could be investigated separately to gain an understanding of the sophistication of the subject (Russell and Snodgrass, 1987; Gartner, 1993; Kim and Yoon, 2003; Li et al., 2010). However, there is an inadequate number of studies that provide a clear understanding of the interrelationship between the cognitive, affective, and conative images. Specifically, it is contended by Gartner (1993) that components were constructed in a hierarchical manner where cognitive images precede affective images. They were also hierarchical with the affective images preceding conative images. This proposition is in line with attitude theory Fishbein and Ajzen (1975) which developed a causal relationship among the cognitive, affective, and conative images. On the other hand, Bagozzi (1992) insisted on the immediate impact of cognitive and affective images upon conative images.

### Hofstede's cultural dimensions

Hofstede's cultural framework, presented in this study, is one approach that is widely used to understand human behaviors from a cross-cultural perspective. It helps identify the most relevant cultural factors to be included in a theoretical framework. Being multi-dimensional, this model proposed employees' cultural values at international enterprises comprised of four dimensions ranging from power distance, individualism-collectivism, masculinity-femininity, to uncertainty avoidance Hofstede (1980). These dimensions are based on Hofstede's empirical investigations of IBM employees in large quantities. These data were collected from more than 70 countries from 1967 to 1973. For optimizing these dimensions, another two dimensions, long-term orientation, and indulgence were integrated into the cultural framework Hofstede and Minkov (2010). Although this framework was an early attempt by Hofstede to provide a theoretical underpinning for cross-cultural study, it remains the most universally utilized cultural framework (Soares et al., 2007).

Hofstede's cultural framework also attracted criticism, among which some are statements that the sample he chose is not representative enough Steenkamp (2001). Others claimed that it was outdated White and Tadesse (2008) and considered it to have insufficient theoretical support (Soares et al., 2007). Moreover, Terlutter et al. (2006) pinpointed (1980, 2001) that unknown values and behaviors in Hofstede's dimension would result in further flaws in his cultural dimensions theory. From a tourism perspective, cultural difference is merely reflective of temporal changes which determine value heterogeneity among citizens within one country (Liu et al., 2018). As a result, various perceptions of Hofstede's cultural dimensions would be formed by people from different cultural backgrounds. Therefore, the dimensions of Hofstede's culture should be proposed in the tourism context to conduct further empirical studies using various samples.

While expounding Hofstede's cultural multidimensions further, scholars who had conducted tourism research (Kirkman et al., 2006) proposed that (1) uncertainty avoidance (Money and Crotts, 2003; Kozak et al., 2007; Reisinger and Crotts, 2009; Quintal et al., 2010; Pan and Truong, 2018) and (2) individualism (Litvin and Kar, 2004; Matzler et al., 2016) were the two cultural dimensions most relevant in tourism studies and cross-cultural tourist behavior (Litvin and Kar, 2004; Litvin et al., 2004; Lam et al., 2012; Matzler et al., 2016; Han et al., 2017). It was also suggested that the interrelationship between the cognitive, affective, and conative images and the incorporation of interdisciplinary constructs was overlooked by previous literature. Therefore, the implementation of the constructs of uncertainty avoidance and individualism was believed to be beneficial to this research model.

Hofstede's theory of cultural multi-dimensions is congruent with the definition of culture in the field of international marketing (Soares et al., 2007) and cross-cultural investigations on tourists (Reisinger and Turner, 2003; e.g., Ng et al., 2007; Wong, 2015). The destination image model was proposed to be linked to Hofstede's uncertainty avoidance and individualism. Given this, the interrelationship between these constructs could be assessed, which might contribute to the successful integration of a theoretical model. Furthermore, the linkage theory of Hofstede's cultural dimensions and the theory of destination image (i.e., cognitive, affective, and conative images) is an umbrella term that covers cognitive, affective, and conative images, including uncertainty avoidance and individualism. Overall, it is hoped that the interrelationship between the constructs of uncertainty avoidance and individualism in this conceptual model, including the cross-cultural approach, will enhance the understanding of tourists' intentions to revisit and recommend.

## Proposition development

### The definition of destination image model and their relationships

According to Crompton (1979), destination image is “*the sum of beliefs, ideas, and impressions that a person has of a destination*” (p.18), whose concept of destination image gained the interest of researchers in the tourism discipline since it is a crucial component of tourists' destination choices (Bonn et al., 2005). Based on this theoretical perspective, destination image is influential for tourists to choose their destinations which is more of a result of how they perceive alternative destinations (Tasci and Gartner, 2007; Chen et al., 2013). Considering this from a practical perspective, the assessment of destination image is identified as a crucial foundation for tourism marketing as it presents images that indicate the perception of pros and cons of a destination from future tourists' standpoints (Baloglu and McCleary, 1999; Tasci and Gartner, 2007).

Extensive works of literature were done in the field of destination image that mainly focused on several themes, including the process of the formation of a destination image (Gartner, 1993; Gallarza et al., 2002). Previous studies in the tourism field (Baloglu and McCleary, 1999; Beerli and Martín, 2004b; Pike and Ryan, 2004; Nadeau et al., 2008) conceptualized the process of how the model of the destination image is established according to attitude theory (Fishbein, 1967; Fishbein and Ajzen, 1975). To elaborate on the attitude theory, attitudes are formed through cognition and affective images, including behavior (Fishbein, 1967; Fishbein and Ajzen, 1975). Scholars in the tourism field (Baloglu and McCleary, 1999; Beerli and Martín, 2004b; Pike and Ryan, 2004; Li et al., 2010; Agapito et al., 2013; Stylidis et al., 2020a,b) frequently conceptualized destination image as three interdependent concepts, namely cognitive, affective, and conative images. To be specific, cognitive image elaborates on potential tourists' perceptions of a destination, affective image elaborated on their attitudes toward the destination, and conative image elaborated on their behavioral intentions of visiting and recommending the destination. Quantitative and qualitative studies concerning destination image recently imply that the cognitive image exercises an impact on the affective image (Ryan and Cave, 2005; Lin et al., 2007; Hyun and O'Keefe, 2012). Regarding tourists' behavioral intentions, scholars found that affective destination image influenced the conative destination image (Li et al., 2010; Agapito et al., 2013; Chew and Jahari, 2014; Hallmann et al., 2015; Fu et al., 2016; Khan et al., 2017).

The components of the conative image are known as tourists' behavioral intentions. According to Oliver (1997), although tourists' behavioral intentions to revisit and recommend are within the scope of conative loyalty, they do not fall under action loyalty. Studies were conducted on revisiting intention as the outcome of an affective situation (Bigné et al., 2001; Kim et al., 2013) since emotions could be the determinant that estimated behavior (Yu and Dean, 2001). More importantly, the aforementioned relationship in the destination image model was verified in previous research. Additionally, affective images have proved to mediate the effects of cognitive images upon behavior intention (Baloglu and McCleary, 1999; Pike and Ryan, 2004; Agapito et al., 2013; Fu et al., 2016). Based on the conceptual and empirical perspectives presented in previous literature, the following propositions are suggested:

Proposition 1: Cognitive image positively influences affective image.

Proposition 2: Affective image positively influences conative intention.

Proposition 2a: Affective image positively influences revisit intention.

Proposition 2b: Affective image positively influences recommendation intention.

Proposition 3: Affective image mediates the effect of cognitive image on conative intention.

Proposition 3a: Affective image mediates the effect of cognitive image on revisit intention.

Proposition 3b: Affective image mediates the effect of cognitive image on recommendation intention.

### The moderating effect of hofstede's cultural dimensions

As an elementary concept, culture manifests individuals' social and consumption behaviors as “a collective programming of the mind which distinguishes one group from another” (Hofstede, 1980, p. 25). This concept is similar to the notion which perceives culture as an integrative mixture of common traits that affect the responses of a group of individuals toward the general environment. Considering these definitions, culture is likely to be embedded in every member of a group and comprises a particular collection of perceptions, beliefs, and behaviors among group members (Cho et al., 2013). Existing studies in the cross-cultural discipline often applied two distinct cultural patterns, namely individualism (Triandis and Gelfand, 1998; Litvin and Kar, 2004; Sivadas et al., 2008) and uncertainty avoidance (Money and Crotts, 2003; Crotts, 2004; Duronto et al., 2005; Reimann et al., 2008). These two typologies from Hofstede's model are clearly defined: uncertainty avoidance is described as the extent to which members of a society feel uncomfortable with uncertainty and ambiguity; and in an individualistic culture, citizens are independent of each other and favor a loosely organized social structure where the emphasis is placed on the care of one's immediate family and self (Hofstede, 1980, 1984). These patterns contribute to an understanding of tourists' behaviors. Similarly, in marketing literature, based on the implementation of Hofstede's cultural framework (1980), individualism and uncertainty avoidance were the two most relevant cultural dimensions.

Individualistic culture has a higher association with private attributes, abilities, beliefs, and characteristics that make an individual unique, special and distinguished from others (Cross et al., 2003). In diverse sectors, individuals originating from English-speaking countries had a higher association with high individualism (Sivadas et al., 2008; Park and Lee, 2009; Cho et al., 2013; Han and Hwang, 2013). Furthermore, as individuals with high individualism often possess strong tendencies for independent thinking, they rarely obtain advice from others. Meanwhile, it was proven through empirical evidence from consumer behavior literature that a buyers' decision-making was significantly influenced by their individualism (Kacen and Lee, 2002), but other studies found that cultural individualism had a moderating effect on consumers' decision-making and behaviors (Iverson, 1997; Crotts and Pizam, 2003; Lee and Lee, 2009; Han and Hwang, 2013). In destination image studies, Litvin and Kar (2004) suggested that individualism was a moderating factor of a destination self-image concept. Individualism was proven to negatively moderate the correlation between self-congruity and behavioral intention (Matzler et al., 2016). Extensive research, conducted on the relationship between cognitive, affective, and conative images, have shed light on tourists' behavioral intentions (conative image) to revisit and recommend destinations (Agapito et al., 2013; Fu et al., 2016). However, the incorporated moderator effects of individualism on cognitive and conative images were omitted from these works, possibly leading to failure in understanding cross-cultural travel behavior. Moreover, travelers who are from individualistic cultures might possess different perspectives on affective and conative images. To provide further insights, the following propositions were suggested:

Proposition 4: The cultural dimension of individualism moderates the relationship between affective image and conative image.

Proposition 4a: The cultural dimension of individualism moderates the relationship between affective image and revisiting intention

Proposition 4b: The cultural dimension of individualism moderates the relationship between the affective image and recommendation intention.

Uncertainty avoidance is a primary Hofstede's cultural multidimension that underpins human judgment and decision-making. It is also a conceptualized feature of risk (Ladbury and Hinsz, 2009). Taking Hofstede's (1980) cultural framework into consideration, uncertainty avoidance mainly highlights the willingness of culture to tolerate the unknown. Cultures could be distinguished based on avoidance of or tolerance to uncertainty (Money and Crotts, 2003). Specifically, the variable of uncertainty avoidance might define the cognitions and behavioral guidance through notable approaches that could also determine whether the variability was cross-cultural or vice versa.

The dimension of uncertainty avoidance is relevant to destination image. Money and Crotts (2003) found that in certain cultures, vacation purchasing decisions were highly influenced by a strong sense of uncertainty avoidance. With respect to tourists' cross-cultural backgrounds, differences between their assessments of destination image were present in their perceptions of uncertainty avoidance (MacKay and Fesenmaier, 2000). Several other studies provided evidence that the cognitive and affective components of the destination image are associated with uncertainty avoidance in the context of cross-cultural travels (Reisinger et al., 2009; Yacout and Hefny, 2015). Furthermore, uncertainty avoidance moderates tourists' satisfaction and behavioral intentions (Reimann et al., 2008; Matzler et al., 2016; Yang et al., 2019a). However, there has been insufficient attention given to this role of uncertainty within cross-cultural perspectives. To provide new insights on this matter, it was presumed that the effects of affective image on the conative image could be enhanced by considering the high relative strength of uncertainty avoidance. Therefore, the following propositions were predicted:

Proposition 5: The cultural dimension of uncertainty avoidance moderates the relationship between affective images and conative images.

Proposition 5a: The cultural dimension of uncertainty avoidance moderates the relationship between affective image and revisit intention.

Proposition 5b: The cultural dimension of uncertainty avoidance moderates the relationship between affective image and recommendation intention.

## Conclusion

### Theoretical contribution

**Figure 1:**
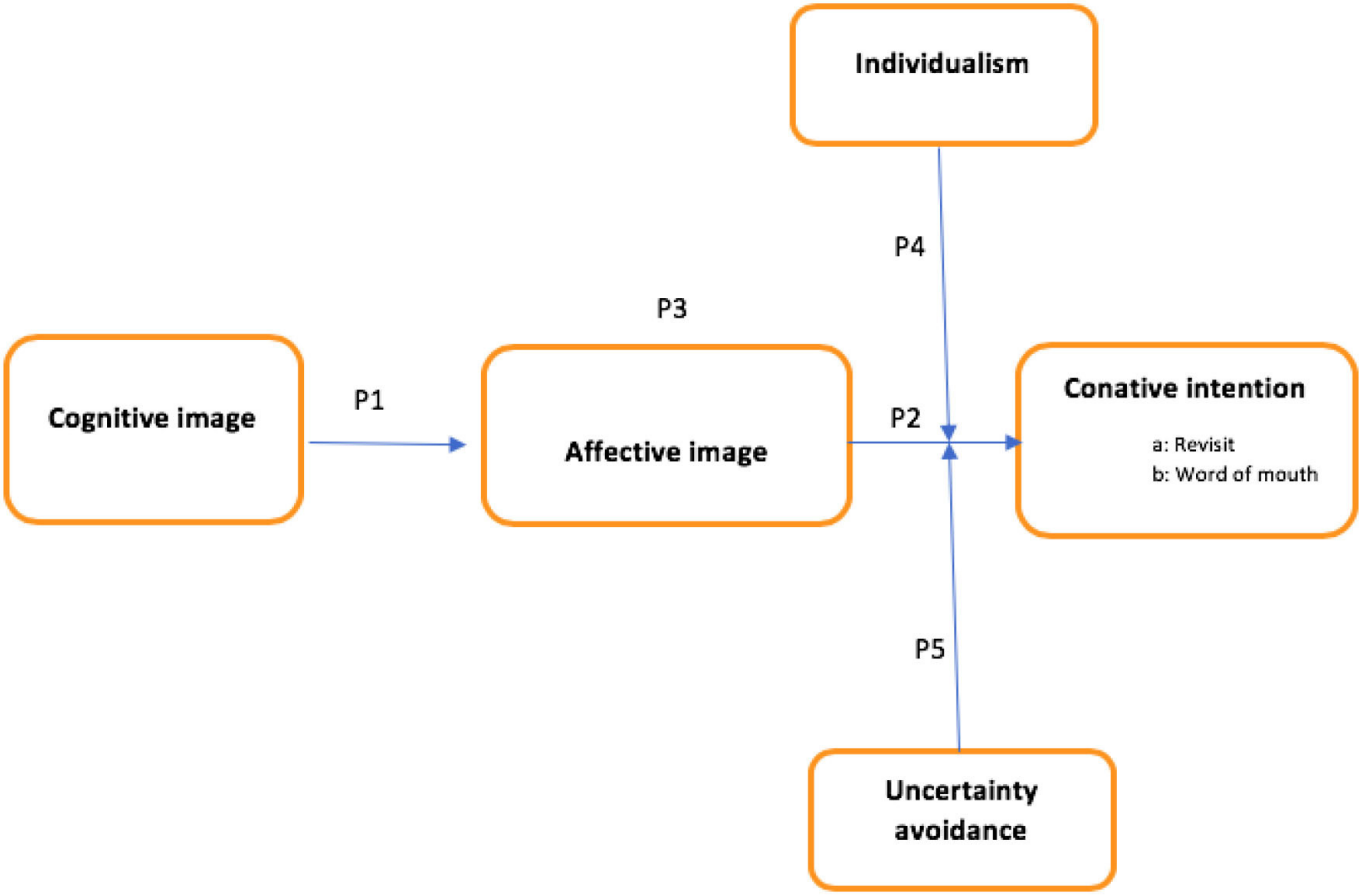
Theoretical framework.

This conceptual paper sheds light on tourism research by developing a theoretical framework for tourists' behavioral intention to revisit a destination. Although empirical data from previous studies have confirmed the image model by exploring the relationship between cognitive image, affective image, and behavior intention in various tourism contexts (Agapito et al., 2013; Stylos et al., 2016; Woosnam et al., 2020; Yang et al., 2021c), the proposed cultural related factors are almost neglected in their conceptualizations. The market internationalization and travel barriers have made it essential to define the construct of culture with different meanings for different landscapes. Hence, the current conceptual paper bridges a gap in prior research by providing a theoretical framework (see Figure 1) that contributes to the body of knowledge in the tourism field.

First, the notable association between uncertainty avoidance, individualism, and behavioral intentions (Matzler et al., 2016; Seo et al., 2018; Yang et al., 2021b, 2022) has been already established; it could be seen that Hofstede's cultural dimensions, including uncertainty avoidance and individualism, play crucial roles in predicting the level of tourists' loyalty. A failure to understand tourists' perceptions of uncertainty avoidance and degree of individualism would result in ineffective marketing. By adopting individualism and uncertainty avoidance, this article also illuminates how tourists across cultures perceive and evaluate their behavioral intention which becomes a crucial issue to determine the success of international tourism. Therefore, by adding uncertainty avoidance and individualism into the framework, this article contributes to the integration of a theoretical model.

Second, this article discusses previous findings about the interaction between cognitive-affective images and conative image in theoretical models (Agapito et al., 2013); however, the existing moderator effect remains to be explored within the domain of destination marketing and destination image. This paper, unrestricted by constructs of direct and indirect associations, introduces a new moderator factor in the relationship model of affective and conative images. Specifically, a more fine-grained description of the interaction between the above-mentioned variables in a theoretical framework was obtained. This article meanwhile investigates destination image with another two constructs (i.e., uncertainty avoidance and individualism) involved to provide an improved explanation based on the perspectives of the cognitive and affective models.

Third, in this article, consideration was placed on an experiential view that could provide more insight into Hofstede's dimensions of individualism and uncertainty avoidance. These insights were produced by merging the theoretical model with the cognitive, affective, and conative images. The proposed connection of these two theories may predict conative image in terms of intention to revisit and recommend. This was followed by the integration of these components into a theoretical framework. Furthermore, it is crucial to highlight that the development of an integrated theoretical framework in this conceptual paper was based on two underlying theories, namely the cognitive, affective, and conative models, and Hofstede's dimensions of individualism and uncertainty avoidance. The merging of these theories contributed to a comprehensive understanding of cross-cultural travel behavior among destination marketing organizations. Considering that individualism and uncertainty avoidance are regarded as the critical components of the hedonic cross-cultural travel experience, this article also offers complimentary values to research on tourism for hedonic consumption.

Last, this conceptual paper also successfully provided new insights into the body of knowledge on tourists' loyalty, specifically in terms of intention to revisit and recommend by investigating the factors of loyalty in the tourism context. Moreover, the moderating roles of individualism and uncertainty avoidance that take place between affective and conative images were introduced. In other words, the positive effect of affective image on conative image is strong for the tourists exhibiting individualistic culture. Also, the positive effect of affective image on conative image is stronger for the tourists exhibiting uncertainty avoidance. As a result, this study has contributed to the conceptualization of the overall destination image model from cross-cultural tourists' perspectives. It also offers notable insights to fully capture the complex subject that revolves around the intent to revisit and recommend a destination among international tourists.

### Practical contribution

Although the proposed theoretical framework has not been tested empirically, several potential practical implications for tourism stakeholders have been offered in this study. One of the implications is the substantial insights provided for destination marketing organizations (DMOs). These insights were derived from observation and analysis of the significance of tourists' perceptions of uncertainty avoidance and individualism. These elements were notable factors that identified tourists' behavioral intentions to revisit and recommend. Moreover, tourism marketers might achieve an improved understanding of a destination's cultural background, which is vital in the implementation of effective marketing strategies.

With the theoretical framework proposed in this study, DMOs would be able to apply effective marketing segmentation and determine the target for potential cross-cultural travelers. Besides, it would be possible to identify a culturally diversified destination with the highest value. Such a destination enables communication and encourages interesting tourism activities, fulfilling the objective of minimizing the cultural difference between the tourism destination and the tourists' home countries. Furthermore, by understanding the relationship between these variables, destination marketers could constantly advertise tourism destinations to potential international tourists. For the managers, this conceptual paper may offer valuable examples of the influence of culture on tourists' behaviors and decision-making processes. These are the factors that assist marketing managers in developing cross-cultural skills and dealing effectively with tourists from diverse cultural backgrounds.

Concepts discussed in this paper provide suggestions for marketing managers to devise appropriate marketing policies to encourage revisits from tourists. In essence, cognitive and affective images of a destination pave the solid foundation for the consideration of alternative products supplied to tourists. Hence, the aforementioned components require serious attention in the development of a positioning strategy for tourism destinations. It should be highlighted that with the relatively unstable nature of destination image, decision-makers may have to constantly observe destination images to adjust their strategic marketing plans.

## Limitations and suggestions for future research

Despite the emphasis on the theoretical and practical contributions of this study, there are some limitations that require to be addressed in future studies. First, this conceptual paper is entirely theoretical; therefore, an empirical test on the framework and the emerging propositions has not been conducted. The second limitation has some connections with the construct of conative image. To be specific, however important attracting tourists' loyalty is to DMOs' successful outcome, marketing practitioners must decide the marketing budget or profits before the investment.

Meanwhile, this article has some weaknesses in the measurement of cultural variables. This article only highlights the variable of culture based on Hofstede's cultural multidimensions, namely individualism and uncertainty avoidance. These dimensions might fail to cover the comprehensive picture of cultural distance. Though this study considers individualism and uncertainty avoidance, it remains questionable whether those two factors can illustrate the complex nature of culture for the sake of the research objective. To address this, future studies are advised to unearth both Hofstede's cultural dimension and other related compositions, such as WVS (World Value Survey) framework and Schwartz's framework (Rokeach, 1973; Inglehart, 1997). Moreover, Yang et al. (2019b) highlighted the importance of introducing other well-formulated cultural variables to estimate how compositions of culture influence the destination selection of international tourists.

This conceptual framework should be viewed with consideration of various destinations as this framework is not necessarily applicable to a single destination. The proposed universal framework is a candidate for further empirical research in cross-country destinations such as China, the US, the UK, and Russia. Tourism destinations of different cultures feature distinguishable characteristics which are worthy of further exploration. Therefore, future studies could introduce this framework as a theoretical basis to explore travel behavior by selecting samples of discrete cultural backgrounds. Differences exist among tourists from different cultures as to what they expect in a destination (Huang and Crotts, 2019). Hence, further explorations of those emerging themes in a cross-cultural travel context would make an interesting and meaningful contribution, which might enhance the generalization of the theoretical framework. This could also provide more insights into the body of tourism literature.

In this conceptual paper, several substantial factors were addressed from theoretical perspectives. Hence, it is recommended that other factors should be considered by future studies that aim to formulate a theoretical framework for predicting tourists' behavioral intentions. Instead of solely implementing the cognitive-affective-conative model, other factors could be incorporated through further application of self-congruity. Notably, this study found that affective image, individualism, and uncertainty avoidance often had a positive impact on conative image. However, it resulted in a discrepancy between tourists' behavioral intentions to revisit and recommend, and their actual behaviors of revisiting and recommendation. As a solution, it is crucial to conduct an empirical test on the interaction mechanism between the conative behavioral intention of revisiting a destination and the actual conative behavior. Similarly, the extension of other related behavioral theories might offer more insightful suggestions.

This conceptual paper has successfully integrated diverse concepts in tourism marketing through the implementation of a theoretical structure underpinned by two existing theories, namely Hofstede's cultural dimensions and the cognitive-affective-conative model. Notably, these theories involve interdisciplinary applications. Furthermore, the test conducted on this theoretical framework suggests a positivist paradigm, a modification of existing scales in the marketing and management areas, and a demand for a quantitative approach for data collection and analysis. This article has introduced a framework with the potential of providing meaningful theoretical and practical implications for academicians and practitioners in the tourism domain.

## Data availability statement

The original contributions presented in the study are included in the article/supplementary material, further inquiries can be directed to the corresponding author.

## Author contributions

SY and JX: conceptualization. YY and JX identified theoretical issue and research gap. SY and SMI: developed theoretical framework. SY: writing—original draft and proofreading. SMI and DL: reviewing, revising, and supervision. YY: project administration. YY, JX, and DL: editing and formatting. All authors contributed to the article and approved the submitted version.

## Funding

This work was supported by Improvement Project of the Basic Research Ability of Young and Middleaged College Teachers in Guangxi (2022KY1305) and Research on Innovative Service Mode of Smart Tourism in the Post-COVID-19 Era.

## Conflict of interest

The authors declare that the research was conducted in the absence of any commercial or financial relationships that could be construed as a potential conflict of interest.

## Publisher's note

All claims expressed in this article are solely those of the authors and do not necessarily represent those of their affiliated organizations, or those of the publisher, the editors and the reviewers. Any product that may be evaluated in this article, or claim that may be made by its manufacturer, is not guaranteed or endorsed by the publisher.
